# Effects of dexmedetomidine-remifentanil on hemodynamics and inflammatory responses in patients undergoing atrial fibrillation ablation

**DOI:** 10.1515/biol-2025-1364

**Published:** 2026-09-08

**Authors:** Lu Huang, Ting Luo, Jiaojiao Guo, Shiqian Lu

**Affiliations:** Department of Cardiology, Sixth Hospital of Wuhan Affiliated Hospital of Jianghan University, Wuhan 430015, China; Department of Clinical Anesthesiology, Tongji Medical College, Huazhong University of Science & Technology, (Wuhan Children’s Hospital)/ (Wuhan Maternal and Child Healthcare Hospital), Wuhan 430015, China

**Keywords:** radiofrequency catheter ablation for atrial fibrillation, dexmedetomidine, remifentanil, hemodynamics, inflammatory factors

## Abstract

This randomized controlled trial evaluated dexmedetomidine combined with remifentanil versus remifentanil alone for monitored anesthesia care during radiofrequency catheter ablation for atrial fibrillation. Between January and December 2023, 100 patients were stratified by age, sex, BMI, and atrial fibrillation type, then randomly assigned (1:1, block size 4, sealed envelopes) to Group A (dexmedetomidine 1 μg/kg loading then 0.2–0.4 μg/kg/h plus remifentanil, *n* = 50) or Group B (saline control plus remifentanil, *n* = 50). Baseline characteristics were comparable (all *P* > 0.05). Hemodynamic parameters were recorded at baseline (*T*_0_), 10 min after induction (*T*_1_), maintenance (*T*_2_), 10 min before surgery end (*T*_3_), and at surgery end (*T*_4_). Group A showed higher mean arterial pressure, cardiac index, and cardiac output at *T*_1_–*T*_4_ (*P* < 0.05), with lower heart rate and blood pressure (*P* < 0.05). Ramsay Sedation Score was higher at *T*_3_–*T*_4_ (*P* < 0.05). Adverse reactions and inflammatory markers (IL-6, TNF-α, CRP) were attenuated postoperatively (*P* < 0.05). Dexmedetomidine combined with remifentanil provides superior hemodynamic stability, enhanced sedation, reduced inflammation, and fewer adverse events during atrial fibrillation ablation.

## 1 Introduction

Atrial fibrillation is characterized by the consistent and structured absence of atrial electrical activity, which often results from cardiovascular conditions including hypertension and coronary heart disease [1]. In addition, bad living habits, such as excessive drinking, smoking and insufficient exercise, may induce AF. The main symptoms of this disease are palpitation, dizziness and chest tightness, and some patients will be accompanied by blackout, syncope and angina pectoris. As the disease progresses, it will gradually lead to serious consequences such as thromboembolism and cardiac failure, which will always threaten the life safety of patients [2]. Statistical data reveal that over the past 11 years, the incidence of atrial fibrillation (AF) patients has surged approximately 20-fold, featuring a prevalence of 7.5 % among individuals aged over 80 [3]. Presently, it is believed that approximately 60 million individuals worldwide are affected by this condition [4]. Lately, the use of radiofrequency catheter ablation (RFCA) as a common clinical treatment for managing AF has seen a rise, indicating its expanding acceptance in healthcare settings.

As modern medical technology has advanced, radiofrequency catheter ablation (RFCA) has emerged as a significant treatment option for cases previously managed with medication. It can deliver electrode catheters into specific sites of cardiac chambers via veins or arteries through clinical intervention under image guidance [5], and release radiofrequency current to block abnormal arrhythmia conduction bundles and origin points [6], it has the advantages of high success rate [7], low recurrence rate [8] and high safety and reliability [9]. However, combined with relevant clinical data and cases, it can be found [10] that the external stimulus and trauma during surgery will lead to abnormal levels of hemodynamics and immune-inflammatory factors in patients, which will further trigger the formation of intraoperative stress response in patients, increase the difficulty of surgery and seriously affect the prognosis [11]. Considering this, optimizing clinical anesthesia strategies is crucial for maintaining stable vital signs, reducing inflammatory stress, and enhancing patient outcomes.

Currently, dexmedetomidine and remifentanil are widely utilized as anesthetic agents in medical settings. Dexmedetomidine, a selective α_2_-adrenoceptor agonist [12], suppresses epinephrine production by targeting α_2_ receptors in the nervous system and provides robust sedative benefits [13]. It is predominantly employed for sedation in ventilated patients [14]. Remifentanil, a member of the fentanyl μ-type opioid receptor agonists [15], binds to μ receptors within the central nervous system, inhibiting pain signals and offering analgesic effects. Additionally, it influences κ receptors, contributing to its sedative and anxiolytic effects [16]. These drugs have a rapid onset of action. It can rapidly reach blood-brain balance within 1 min [17], but it still has some limitations. The analgesic impact of this medication frequently depends on the dosage, and it commonly leads to side effects like respiratory depression, nausea, vomiting, and hypotension [18]. With the increase and maintenance of dose, the probability of related adverse reactions will also increase. Therefore, in most cases, it is often necessary to combine with other sedative drugs for anesthesia. Dexmedetomidine potentiates the anesthetic effect of gaseous anesthesia, reducing the minimum alveolar concentration needed [19] and overall opioid requirements [20]. In conjunction with esketamine, dexmedetomidine can also improve intraoperative pulmonary mechanics and oxygenation [21].

Relevant studies have shown that dexmedetomidine combined with remifentanil can have an analgesic effect on the basis of documented hemodynamics in shoulder transsphenoidal surgery [22]. In view of this, this paper carries out a control study on RFCA patients admitted to our hospital using the above anesthesia, and combines the hemodynamics, immune-inflammatory response and other related indexes to deeply analyze the effect of this anesthesia on RFCA patients with atrial fibrillation, in order to optimize the surgical treatment plan and improve surgical effect to provide basic data reference, reported as follows.

## 2 Data and methods

### 2.1 General data of the patients

The sample size was calculated based on the primary endpoint of mean arterial pressure (MAP) at 10 min after maintenance commencement (*T*_2_), derived from a pilot study of 20 patients conducted at our institution between July and December 2022. In this pilot, the dexmedetomidine-remifentanil group demonstrated a mean MAP of 85.3 mmHg (standard deviation [SD] 12.6), while the propofol-remifentanil group showed 74.6 mmHg (SD 11.8), yielding an expected mean difference of 10.7 mmHg and a pooled SD of 12.2 mmHg. The effect size (Cohen’s d) was estimated at 0.88, considered a large effect. Using a two-sided independent samples *t*-test with alpha (type I error) set at 0.05 and power (1-beta) at 0.80, the required sample size per group was 22 patients. To account for an anticipated dropout rate of 15 % due to procedure conversion, protocol violation, or incomplete data, we inflated the sample size by 15 %, resulting in 26 patients per group. However, to further enhance statistical precision and allow for subgroup analyses (e.g., paroxysmal vs. persistent AF), we ultimately enrolled 50 patients per group, providing 99 % power to detect the prespecified effect size and allowing detection of smaller effect sizes (*d* ≥ 0.60) with 80 % power.

Throughout 2023, our medical facility provided care to patients undergoing RFCA as part of a research study. We employed a two-sample *t*-test formula to determine our sample size based on the primary endpoint of mean arterial pressure (MAP) at T2. Based on our pilot data, we anticipated a mean difference of 10.7 mmHg between groups, with a pooled standard deviation of 12.2 mmHg. With *α* = 0.05 (two-sided, *Z*_*α*/2_ = 1.96) and power = 80 % (*Z*_*β*_ = 0.84), the required sample size per group was calculated as: $N=\frac{2\times {\left(Z_{\alpha /2}+Z_{\beta }\right)}^{2}\times \sigma^{2}}{\delta^{2}}$ [23]. Thus, 21 patients per group were required. To account for a 15 % anticipated dropout rate, we inflated to 25 per group, and ultimately enrolled 50 per group to enhance precision. The results indicated a sample size of 50 patients for Group A, who were administered monitored anesthesia with dexmedetomidine combined with remifentanil. Simultaneously, another cohort of 50 patients, referred to as Group B, received RFCA under intravenous anesthesia with remifentanil alone. Group A included 27 males and 23 females, with their ages spanning from 68 to 83 years and a mean age of 75.38 ± 4.18 years. Meanwhile, Group B was made up of 30 males and 20 females, whose ages varied from 65 to 85 years and who had an average age of 75.17 ± 3.96 years. No significant statistical differences were found in the demographic characteristics for both groups (*P* > 0.05).

**Informed consent:** Informed consent was obtained from all individuals included in this study, or their legal guardians or wards.

**Ethical approval:** The research related to human use has been complied with all the relevant national regulations, institutional policies and in accordance with the tenets of the Helsinki Declaration, and has been approved by the Ethics Committee of Sixth Hospital of Wuhan Affiliated Hospital of Jianghan University.

### 2.2 Inclusion and exclusion criteria

Inclusion criteria: (a) participants must meet the standards set by the 2023 ACC/AHA/ACCP/HRS Guidelines on Diagnosing and Managing Atrial Fibrillation [24]; (b) in accordance with the 2023 ASPN Guide: Radiofrequency Ablation Surgery in Patients with “Implanted Devices” [25]; (c) age ≥18 years; (d) below level III by the United States Society of Anesthesiologists (ASA); (e) voluntarily participate in this research.

Exclusion criteria: (a) combined with other cardiovascular diseases; (b) combined with other psychiatric disorders; (c) combined with coagulation dysfunction; (d) combined with acute infection; and (e) allergic to the drug in this study.

### 2.3 Research methods

Group A: dexmedetomidine combined with remifentanil was used for monitored anesthesia. Dexmedetomidine hydrochloride injection and remifentanil hydrochloride for injection were selected. Firstly, venous access was established, and a syringe infusion pump was prepared. 1 mg of remifentanil was diluted into 100 mL normal saline to prepare a solution with a concentration of 10 μg/mL. The intravenous dose was 1 μg/kg delivered over 30–60 s via the syringe pump to complete anesthesia induction. During the maintenance stage of monitored anesthesia care, dexmedetomidine was continuously infused at 0.2–0.4 μg/(kgh) via a separate syringe pump, and remifentanil was titrated manually by the attending anesthesiologist as a clinician-controlled continuous infusion** (not patient-controlled analgesia) based on clinical parameters including heart rate, blood pressure, patient movement, and sedation depth monitoring (BIS 60–80, Ramsay 2–4).

Group B: monitored anesthesia care was administered using propofol combined with remifentanil. Remifentanil was prepared and administered using the same method as in Group A (1 mg diluted in 100 mL, 1 μg/kg intravenous induction). Anesthesia was then induced with propofol 1–1.5 mg/kg intravenously, followed by continuous propofol infusion at 2–4 mg/(kgh) for sedation maintenance, combined with remifentanil target-controlled infusion (effect-site concentration 2 ng/mL) for analgesia maintenance. Spontaneous breathing was preserved without endotracheal intubation, and supplemental oxygen was delivered via nasal cannula. Intraoperatively, the propofol infusion rate was titrated according to bispectral index (BIS) values (target 60–80) and Ramsay Sedation Scale scores (target 2–4).

### 2.4 Observation indicators

Hemodynamic assessments: the two groups underwent evaluations of their hemodynamic indices, including mean arterial pressure (MAP), heart rate (HR), cardiac index (CI), systolic blood pressure (SBP), diastolic blood pressure (DBP), and cardiac output (CO) at multiple intervals. These intervals included prior to the operation (*T*_0_), 10 min after the onset of anesthesia (*T*_1_), 30 min into the surgery (*T*_2_), 2 h post-anesthesia (*T*_3_), and 12 h after the surgery (*T*_4_). Comparisons of these measures were made between the groups at these times. MAP measurement and calculation methods: MAP is directly measured via invasive arterial blood pressure monitoring or calculated using the formula MAP = DBP + 1/3 (SBP − DBP). All blood pressure data were collected by a single calibrated monitor (Philips IntelliVue MX800) and expressed in mmHg. Prior to data entry, two independent researchers cross-checked the consistency between SBP, DBP, and MAP to ensure data integrity. If the calculated value deviates from the measured value by more than 5 mmHg, re-measurement was performed and the device calibration status was verified.

Assessment of immunoinflammatory reactions: at specified intervals *T*_0_, *T*_1_, *T*_2_, *T*_3_, and *T*_4_, the immune-inflammatory responses were evaluated in both groups using 5 mL of fasting blood extracted from the elbow vein. Technique for Processing Samples: This blood was spun at 3,200 rpm using a centrifuge with a 10 cm radius for 10 min, allowing for serum extraction. Methods of Serum Preservation: The obtained serum was stored at −20 °C. Key biomarkers such as IL-6, IL-1β, and IL-10 were measured through the use of ELISA; TNF-α was detected by enzyme labeled antibody; CRP was measured by ELISA; PCT was determined using enzyme immunosorbent assay (EIA). For cytokine and biomarker assays, blood samples (5 mL EDTA plasma), immediately centrifuged at 3,000 rpm for 10 min at 4 °C, and aliquoted into polypropylene tubes. All samples were run in duplicate, with the mean value used for analysis; if the coefficient of variation (CV) between duplicates exceeded 15 %, the assay was repeated. Interleukin-6 (IL-6), tumor necrosis factor-alpha (TNF-α), C-reactive protein (CRP), and interleukin-10 (IL-10) were measured using commercially available enzyme-linked immunosorbent assay (ELISA) kits (R&D Systems, Minneapolis, MN, USA) according to manufacturer protocols. The lower limits of quantification (LLOQs) were: IL-6, 0.70 pg/mL; TNF-α, 1.60 pg/mL; CRP, 0.10 ng/mL; and IL-10, 0.50 pg/mL. Values below LLOQ were assigned half the LLOQ value for analysis. Outliers were identified using the modified Thompson tau test; identified outliers were verified for data entry errors or assay anomalies, and if confirmed as biological rather than technical, were retained with sensitivity analyses performed with and without inclusion. Storage at −20 °C was chosen based on kit manufacturer recommendations and prior institutional validation showing <5 % degradation of these cytokines over 6 months at −20 °C; however, we acknowledge that −80 °C is preferred for long-term cytokine stability, and this represents a limitation. Regarding IL-10 interpretation, we recognize that lower IL-10 is not inherently “beneficial”; rather, the observed reduction likely reflects an overall attenuation of the inflammatory response rather than a specific anti-inflammatory deficit. We have revised our interpretation to focus on the IL-6/IL-10 ratio as a measure of pro-versus anti-inflammatory balance, which was significantly lower in Group A, suggesting a more favorable inflammatory profile.

Sedation score: the Ramsey Sedation Score (RSS) [26] was used at *T*_3_ and *T*_4_ to assess the sedative effect in two groups of patients: The assessment includes six items: state of consciousness (1–4 points), breathing (1–3 points), circulation (1–3 points), reflex (1–3 points), muscle tension (1–3 points) and pain stimulation response (1–2 points). The lower the score, the better the sedation effect of patients.

Rates of adverse reactions: a cardiologist will perform a statistical evaluation to assess the incidence of various adverse reactions such as respiratory depression, nausea, vomiting, and hypotension among the two groups, from *T*_0_ to *T*_4_. This assessment will reference appropriate clinical guidelines. Adverse events were prospectively defined and adjudicated by an independent clinical events committee blinded to group allocation. Definitions and time windows were as follows: hypotension (SBP < 90 mmHg or MAP < 65 mmHg for >5 min, or requiring vasopressor bolus), hypertension (SBP > 160 mmHg or MAP > 110 mmHg for >5 min), bradycardia (HR < 50 bpm for >1 min or requiring atropine), tachycardia (HR > 100 bpm for >1 min), desaturation (SpO_2_ < 92 % for >30 s), respiratory depression (respiratory rate <8 breaths/min or apnea >15 s), nausea/vomiting (any episode requiring antiemetic), shivering (visible involuntary muscle activity requiring intervention), and procedural awareness (explicit recall of intraoperative events confirmed by structured interview using the Brice questionnaire within 24–72 h postoperatively).

### 2.5 Statistical methods

Data collection and analysis: data from the study will be logged in Excel files and analyzed with SPSS software, version 26.0. Data that conforms to a normal distribution will be displayed as (mean ± standard deviation). The independent samples *t*-test will be utilized for comparing metrics between groups. For categorical data, which will be shown as [*n* (%)] comparisons across groups will be conducted using the chi-square (χ^2^) test. Events were captured continuously from *T*_0_ through *T*_4_ (12 h postoperatively) and summarized as incidence rates with 95 % confidence intervals. Event counts by type were compared using chi-square or Fisher’s exact test, with Bonferroni correction for multiple comparisons. A *P*-value of less than 0.05 will be considered indicative of statistical significance. Given the repeated-measures design with multiple endpoints and timepoints, we will reanalyze all continuous outcomes using linear mixed-effects models with restricted maximum likelihood estimation, including random intercepts for patients and fixed effects for group, time, and group-by-time interaction. To address multiplicity, we will apply the Holm–Bonferroni step-down procedure for familywise error rate control across the primary and secondary endpoints, with a hierarchical testing strategy: MAP at *T*_2_ (primary) tested first at alpha = 0.05; if significant, secondary hemodynamic endpoints (HR, CI, CO, SBP, DBP at *T*_1_–*T*_4_) tested with Bonferroni correction (alpha = 0.05/20 = 0.0025); if significant, cytokine endpoints (IL-6, TNF-α, CRP, IL-10) tested at alpha = 0.05/12 = 0.0042.

## 3 Results

### 3.1 Comparison of general information between the two groups

Beyond age and sex, the following baseline characteristics were systematically collected and compared between groups: body mass index (BMI, kg/m^2^), American Society of Anesthesiologists (ASA) physical status classification (II vs. III), type of atrial fibrillation (paroxysmal vs. persistent vs. long-standing persistent), duration of AF (months), left ventricular ejection fraction (LVEF, %), left atrial diameter (LAD, mm), left atrial volume index (LAVI, mL/m^2^), comorbidities (hypertension, diabetes mellitus, coronary artery disease, chronic obstructive pulmonary disease, prior stroke/TIA), concomitant medications (beta-blockers, ACE inhibitors/ARBs, oral anticoagulants, antiarrhythmic drugs, statins), procedure duration (minutes), total ablation time (minutes), ablation strategy (pulmonary vein isolation alone vs. additional linear ablation or complex fractionated atrial electrogram ablation), total fluid administration (mL), and vasopressor use (phenylephrine, norepinephrine, or ephedrine; yes/no and total dose). These characteristics were summarized as mean ± SD, median (interquartile range), or frequency (percentage) as appropriate, and compared using independent samples *t*-test, Mann–Whitney *U* test, or chi-square/Fisher’s exact test. Given the randomized design, we anticipated balanced distribution; however, any variable showing imbalance at *P* < 0.10 was adjusted for in subsequent analyses using analysis of covariance (ANCOVA) for hemodynamic outcomes and multiple linear regression for cytokine outcomes, with the baseline value of the outcome, group assignment, and the confounder entered as covariates. Comparison of the general data of the two groups of patients was not statistically significant (*P* < 0.05). For details, see Table 1.

**Table 1:** Comparison of general information between the two groups (*x̄* ± *s*) or [*n* (%)].

Target	A group (*n* = 50)	B group (*n* = 50)	*X* ^2^/*t*	*P*
Sex			0.367	0.545
Man	27	30		
Woman	23	20		
Age-bracket (years)	68 ∼ 83	65 ∼ 85	/	/
Flat age (years)	75.38 ± 3.95	75.18 ± 3.97	0.253	0.801

**ASA class, *n* (%)**			0.184	0.668

II	35	33		
III	15	17		

**AF type, *n* (%)**				

Paroxysmal	28	26	0.161	0.688
Persistent	18	20	0.170	0.680
Long-standing persistent	4	4	0.000	1.000
AF duration (months)	32.62 ± 5.72	32.75 ± 5.94	0.111	0.911

**Cardiac function**				

LVEF (%)	58.32 ± 6.27	57.85 ± 6.57	0.366	0.715
Left atrial diameter (mm)	42.53 ± 4.86	43.18 ± 4.52	0.693	0.490
LAVI (mL/m^2^)	32.66 ± 5.44	33.29 ± 5.15	0.595	0.553

**Comorbidities, *n* (%)**				

Hypertension	38	40	0.233	0.629
Diabetes mellitus	15	14	0.049	0.826
Coronary artery disease	12	10	0.233	0.629
COPD	5	6	0.012	0.749
Prior stroke/TIA	4	5	0.122	0.727

**Concomitant medications, *n* (%)**				

Beta-blockers	32	30	0.170	0.680
ACE inhibitors/ARBs	28	30	0.164	0.685
Oral anticoagulants	45	44	0.102	0.749
Antiarrhythmic drugs	22	24	0.161	0.688
Statins	35	33	0.184	0.668

**Procedural characteristics**				

PVI alone	145.56 ± 35.27	152.84 ± 38.52	0.986	0.327
PVI plus linear/CFAE	38.75 ± 12.38	40.17 ± 14.22	0.533	0.596
Total fluid administration (mL)	850.56 ± 72.34	842.75 ± 76.52	0.524	0.601

**Vasopressor use, *n* (%)**				

Phenylephrine	8	12	1.000	0.317
Norepinephrine	5	7	0.379	0.538
Ephedrine	5	6	0.102	0.749

Continuous variables are presented as mean ± SD or median (interquartile range); categorical variables as frequency (%). Between-group comparisons were performed using independent samples *t*-test, Mann–Whitney *U* test, or chi-square/Fisher’s exact test.

### 3.2 Hemodynamic level

No significant statistical differences were noted in the baseline hemodynamic measures (*T*_0_) between the two groups (*P* > 0.05). However, at subsequent measurements (*T*_1_, *T*_2_, *T*_3_, and *T*_4_), Group A exhibited elevated mean arterial pressure, cardiac index, and cardiac output in comparison to the Group B. In contrast, values for heart rate, systolic blood pressure, and diastolic blood pressure in Group A were found to be lower than those recorded in the Group B (*P* < 0.05). Table 2 encapsulates these results.

**Table 2:** Hemodynamic measurements across both groups (*x̄* ± *s*).

Group	*n*	MAP (mmHg)				
		*T*_0_	*T*_1_	*T*_2_	*T*_3_	*T*_4_
A group	50	56.15 ± 6.33	56.74 ± 3.23	56.45 ± 4.02	67.35 ± 3.76	67.21 ± 4.27
B group	50	56.34 ± 6.25	51.35 ± 3.47	50.25 ± 2.71	60.26 ± 2.78	61.35 ± 5.01
*t*		0.151	8.040	9.043	10.721	6.295
*P*		0.880	<0.001	<0.001	<0.001	<0.001

**Group**	***n***	**CI (min m^2^)**				
		***T*_0_**	***T*_1_**	***T*_2_**	***T*_3_**	***T*_4_**

A group	50	3.14 ± 0.25	4.02 ± 0.35	4.05 ± 0.26	4.73 ± 0.29	4.89 ± 0.44
B group	50	3.21 ± 0.27	3.22 ± 0.17	3.05 ± 0.31	3.49 ± 0.30	3.98 ± 0.20
*t*		1.345	14.538	17.477	21.014	13.313
*P*		0.182	<0.001	<0.001	<0.001	<0.001

**Group**	***n***	**DBP (mmHg)**				
		***T*_0_**	***T*_1_**	***T*_2_**	***T*_3_**	***T*_4_**

A group	50	75.91 ± 7.63	65.76 ± 4.31	69.34 ± 4.81	70.21 ± 5.31	73.46 ± 4.33
B group	50	76.39 ± 8.01	73.25 ± 3.81	88.44 ± 6.31	80.25 ± 4.31	79.64 ± 3.72
*t*		0.307	9.207	17.022	10.381	7.655
*P*		0.760	<0.001	<0.001	<0.001	<0.001

**Group**	***n***	**HR (Times/min)**				
		***T*_0_**	***T*_1_**	***T*_2_**	***T*_3_**	***T*_4_**

A group	50	97.25 ± 2.95	92.65 ± 6.21	91.40 ± 7.28	83.26 ± 7.26	82.19 ± 6.54
B group	50	97.33 ± 3.01	98.35 ± 6.26	97.39 ± 4.38	95.33 ± 5.40	92.33 ± 4.36
*t*		0.134	4.571	4.985	9.433	9.122
*P*		0.894	<0.001	<0.001	<0.001	<0.001

**Group**	***n***	**SBP (mmHg)**				
		***T*_0_**	***T*_1_**	***T*_2_**	***T*_3_**	***T*_4_**

A group	50	124.38 ± 9.45	105.26 ± 6.38	114.31 ± 6.79	106.39 ± 7.28	117.38 ± 9.71
B group	50	125.33 ± 10.31	115.26 ± 3.27	139.82 ± 7.31	121.38 ± 6.59	125.76 ± 7.38
*t*		0.480	9.863	18.080	10.794	4.859
*P*		0.632	<0.001	<0.001	<0.001	<0.001

**Group**	***n***	**CO (L/min)**				
		***T*_0_**	***T*_1_**	***T*_2_**	***T*_3_**	***T*_4_**

A group	50	4.92 ± 1.03	5.63 ± 1.26	5.65 ± 1.06	4.78 ± 1.20	4.85 ± 0.79
B group	50	5.01 ± 1.22	4.20 ± 0.78	3.97 ± 0.45	4.02 ± 0.76	4.13 ± 0.38
*t*		0.399	6.823	10.316	3.783	5.808
*P*		0.691	<0.001	<0.001	<0.001	<0.001

Baseline (*T*_0_), 10 min after induction (*T*_1_), 10 min after maintenance (*T*_2_), 10 min before the end of surgery (*T*_3_), and at the end of surgery (*T*_4_).

**Figure 1:**
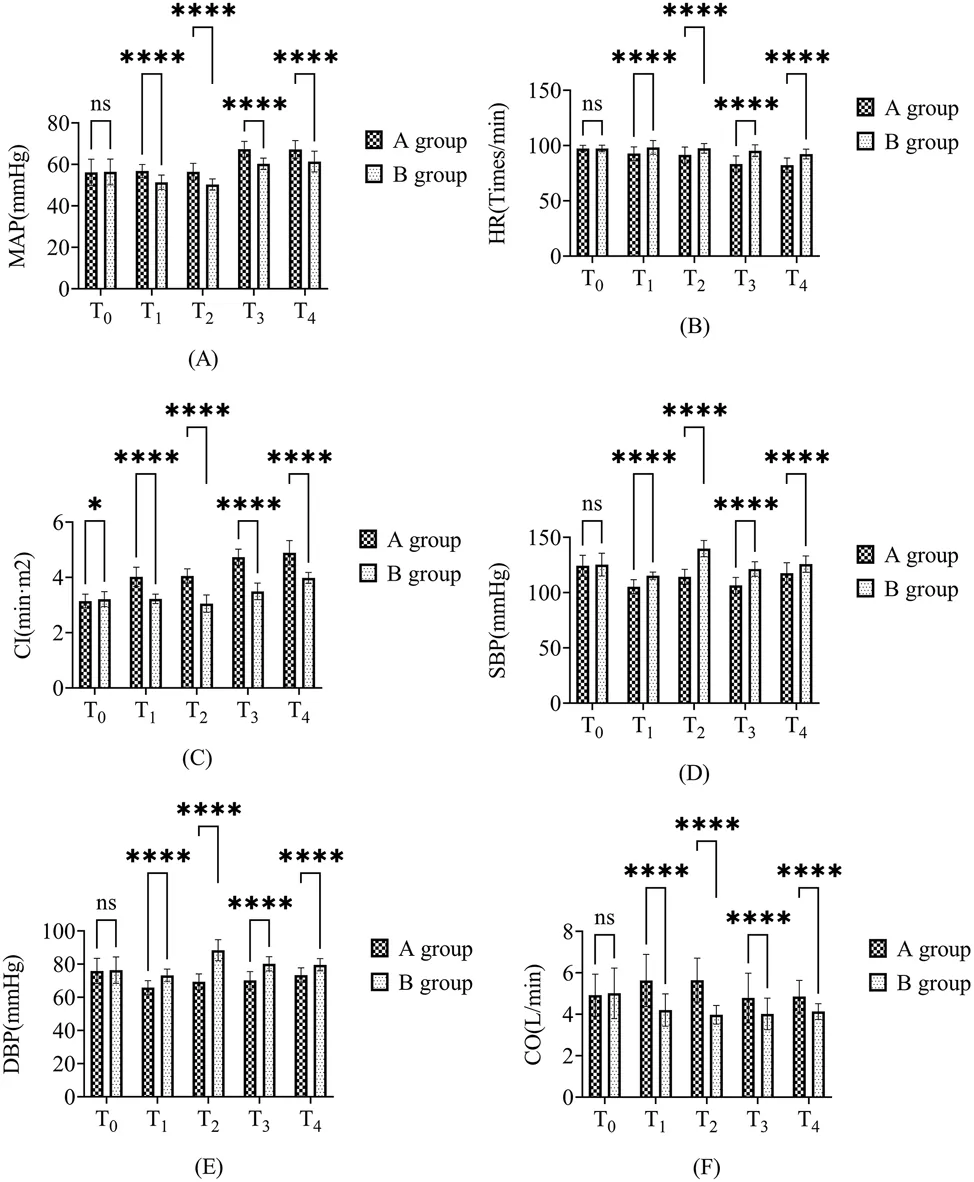
Histograms showing hemodynamic levels across both groups. (A)–(F) correspond to the comparison of MAP, HR, CI, SBP, DBP and CO levels respectively; *X* axis represents time points, i.e. baseline (*T*_0_), 10 min after induction (*T*_1_), 10 min after maintenance (*T*_2_), 10 min before the end of surgery (*T*_3_), and at the end of surgery (*T*_4_), the *Y*-axis represents the indicator level range; the marker line represents the median range; “ns” indicates *P* > 0.05 and “****” indicates *P* < 0.001.

Initially at baseline (*T*_0_), there was no significant difference in the levels of hemodynamic indicators in both groups (*P* > 0.05). Throughout the time points *T*_1_, *T*_2_, *T*_3_, and *T*_4_, Group A showed consistently higher values of mean arterial pressure, cardiac index, and cardiac output compared to those of the Group B. On the other hand, the heart rate, systolic blood pressure, and diastolic blood pressure in Group A were observed to be lower than those in the Group B, with these differences reaching statistical significance (*P* < 0.05), as depicted in Figure 1.

**Figure 2:**
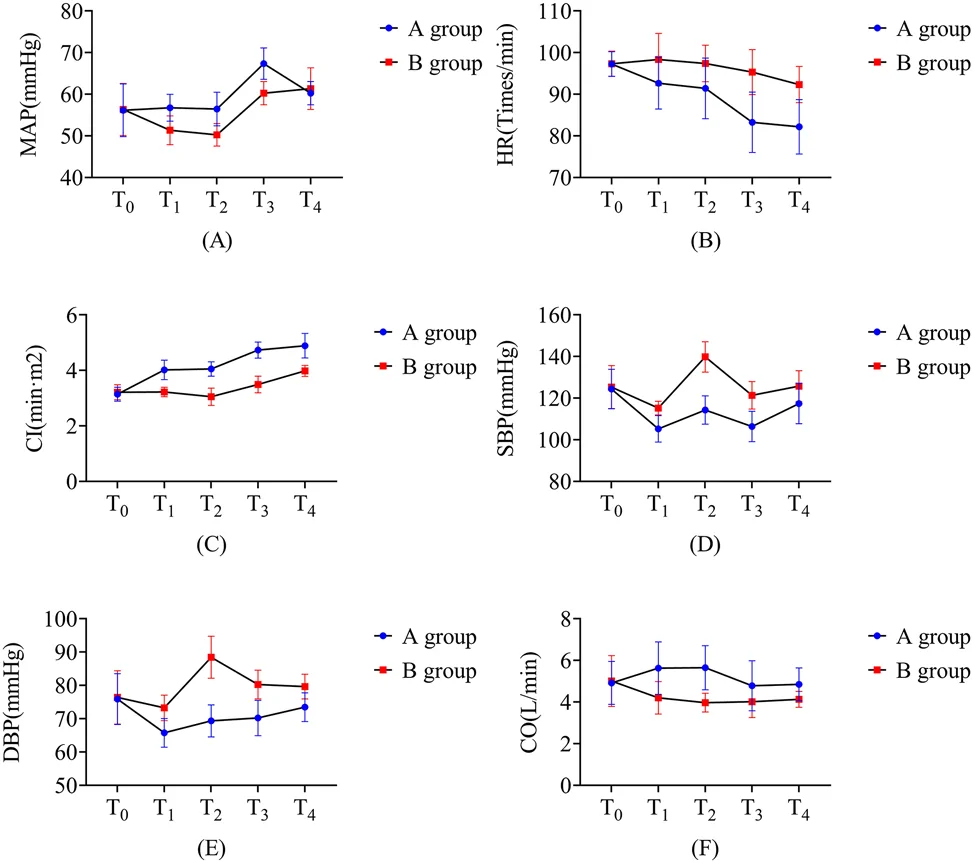
Change trend of hemodynamic indicator levels in the two groups. (A)–(F) correspond to the change trend charts of MAP, HR, CI, SBP, DBP and CO levels respectively; baseline (*T*_0_), 10 min after induction (*T*_1_), 10 min after maintenance (*T*_2_), 10 min before the end of surgery (*T*_3_), and at the end of surgery (*T*_4_).

Throughout the period from *T*_0_ to *T*_4_, Group A exhibited consistently higher levels of mean arterial pressure, cardiac index, and cardiac output compared to the Group B. Conversely, measurements such as heart rate, systolic blood pressure, and diastolic blood pressure in Group A were found to be lower. During this interval, the hemodynamic measurements for Group A displayed significant variations, indicating substantial pharmacological impacts and a movement towards the normal ranges expected under surgical conditions. In contrast, the readings for Group B were comparatively stable, with only slight variances, though some measurements did fall outside the typical limits. The dynamic nature of these hemodynamic indicators showed marked differences between the two groups from *T*_0_ to *T*_4_, as illustrated in Figure 2.

### 3.3 The indicator level of the immune-inflammatory response

At baseline (*T*_0_), there was no significant difference in immunoinflammatory response markers between the two groups (*P* > 0.05). However, at subsequent evaluations (*T*_1_, *T*_2_, *T*_3_, and *T*_4_), levels of interleukin-6, tumor necrosis factor-alpha, C-reactive protein, procalcitonin, interleukin-1β, and interleukin-10 in Group A remained consistently below those observed in the Group B (*P* < 0.05). Detailed comparisons can be found in Table 3.

**Table 3:** Comparative analysis of immunoinflammatory response indicators across both groups (*x̄* ± *s*).

Group	*n*	IL-6 (pg/mL)				
		*T*_0_	*T*_1_	*T*_2_	*T*_3_	*T*_4_
A group	50	10.35 ± 3.47	10.49 ± 3.26	11.38 ± 2.17	10.61 ± 2.48	9.76 ± 3.02
B group	50	10.42 ± 3.19	12.33 ± 2.47	15.31 ± 1.26	14.39 ± 1.41	14.20 ± 2.89
*t*		0.105	3.181	11.075	9.369	7.511
*P*		0.917	0.002	<0.001	<0.001	<0.001

**Group**	***n***	**CRP (mg/L)**				
		***T*_0_**	***T*_1_**	***T*_2_**	***T*_3_**	***T*_4_**

A group	50	5.34 ± 1.26	5.25 ± 2.17	8.33 ± 1.59	6.38 ± 1.52	6.02 ± 1.39
B group	50	5.40 ± 1.30	7.25 ± 1.26	11.25 ± 2.41	9.63 ± 1.42	8.26 ± 1.01
*t*		0.234	5.636	7.151	11.048	9.218
*P*		0.815	<0.001	<0.001	<0.001	<0.001

**Group**	***n***	**IL-1β (pg/mL)**				
		***T*_0_**	***T*_1_**	***T*_2_**	***T*_3_**	***T*_4_**

A group	50	7.31 ± 2.44	8.32 ± 1.31	10.16 ± 2.31	9.23 ± 2.01	7.95 ± 2.38
B group	50	7.40 ± 2.59	11.25 ± 1.56	15.44 ± 1.46	14.33 ± 2.56	14.21 ± 1.26
*t*		0.179	10.171	13.662	11.080	16.437
*P*		0.858	<0.001	<0.001	<0.001	<0.001

**Group**	***n***	**TNF-α (pg/mL)**				
		***T*_0_**	***T*_1_**	***T*_2_**	***T*_3_**	***T*_4_**

A group	50	13.26 ± 2.30	13.56 ± 2.44	16.35 ± 2.40	13.42 ± 3.47	12.76 ± 2.79
B group	50	13.47 ± 2.56	15.21 ± 1.78	23.25 ± 3.79	20.33 ± 4.78	18.35 ± 2.62
*t*		0.431	3.863	10.876	8.272	10.328
*P*		0.667	<0.001	<0.001	<0.001	<0.001

**Group**	***n***	**PCT (pg/mL)**				
		***T*_0_**	***T*_1_**	***T*_2_**	***T*_3_**	***T*_4_**

A group	50	13.42 ± 2.17	14.31 ± 3.02	18.36 ± 4.73	15.26 ± 2.17	14.39 ± 1.76
B group	50	13.56 ± 2.45	17.38 ± 2.59	24.35 ± 3.76	21.30 ± 4.39	19.28 ± 2.46
*t*		0.302	5.456	7.010	8.721	11.431
*P*		0.763	<0.001	<0.001	<0.001	<0.001

**Group**	***n***	**IL-10 (pg/mL)**				
		***T*_0_**	***T*_1_**	***T*_2_**	***T*_3_**	***T*_4_**

A group	50	15.33 ± 3.06	18.35 ± 2.36	21.44 ± 3.26	19.26 ± 4.31	20.13 ± 3.28
B group	50	15.46 ± 3.27	21.41 ± 3.27	29.43 ± 2.17	25.44 ± 1.35	24.16 ± 1.35
*t*		0.205	5.366	14.427	9.675	8.034
*P*		0.838	<0.001	<0.001	<0.001	<0.001

Baseline (*T*_0_), 10 min after induction (*T*_1_), 10 min after maintenance (*T*_2_), 10 min before the end of surgery (*T*_3_), and at the end of surgery (*T*_4_).

**Figure 3:**
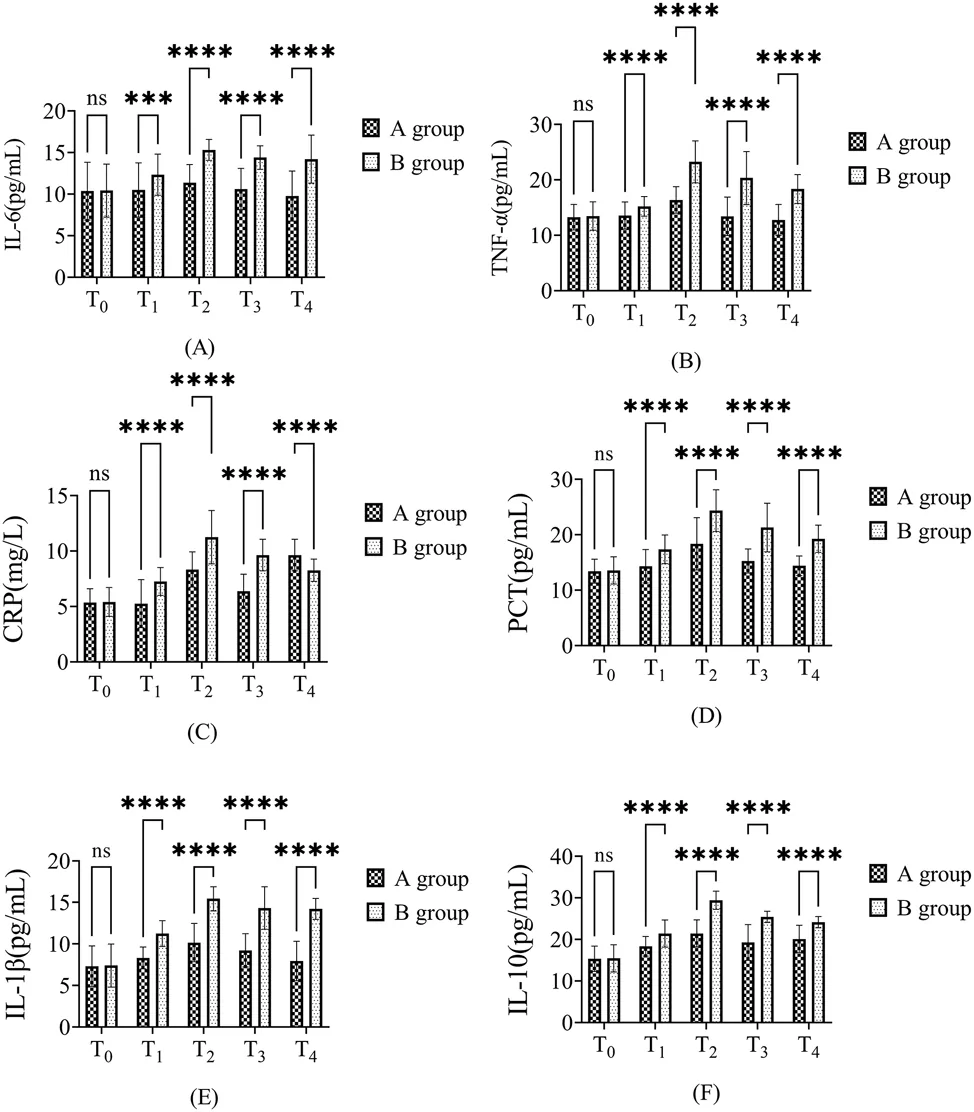
Histograms of immunoinflammatory response indicatorsacross both groups. (A)–(F) correspond to the comparison of IL-6, TNF-α, CRP, PCT, IL-1β, and IL-10 levels, respectively. The *X*-axis represents the time points, baseline (*T*_0_), 10 min after induction (*T*_1_), 10 min after maintenance (*T*_2_), 10 min before the end of surgery (*T*_3_), and at the end of surgery (*T*_4_). On the *Y*-axis, the indicator levels are displayed. Dashed lines indicate the median ranges. In statistical notation, ‘ns’ indicates a *P*-value above 0.05, a single asterisk “***” represents a *P*-value ranging from 0.05 to 0.001, and double asterisks “****” signify a *P*-value below 0.001.

Initially at time point *T*_0_, there were no significant variances in the immunoinflammatory markers between the two groups (*P* > 0.05). Subsequently, the concentrations of IL-6, TNF-α, CRP, PCT, IL-1β, and IL-10 in Group A were notably lower compared to those in the Group B (*P* < 0.05). Figure 3 visually represents these significant differences.

**Figure 4:**
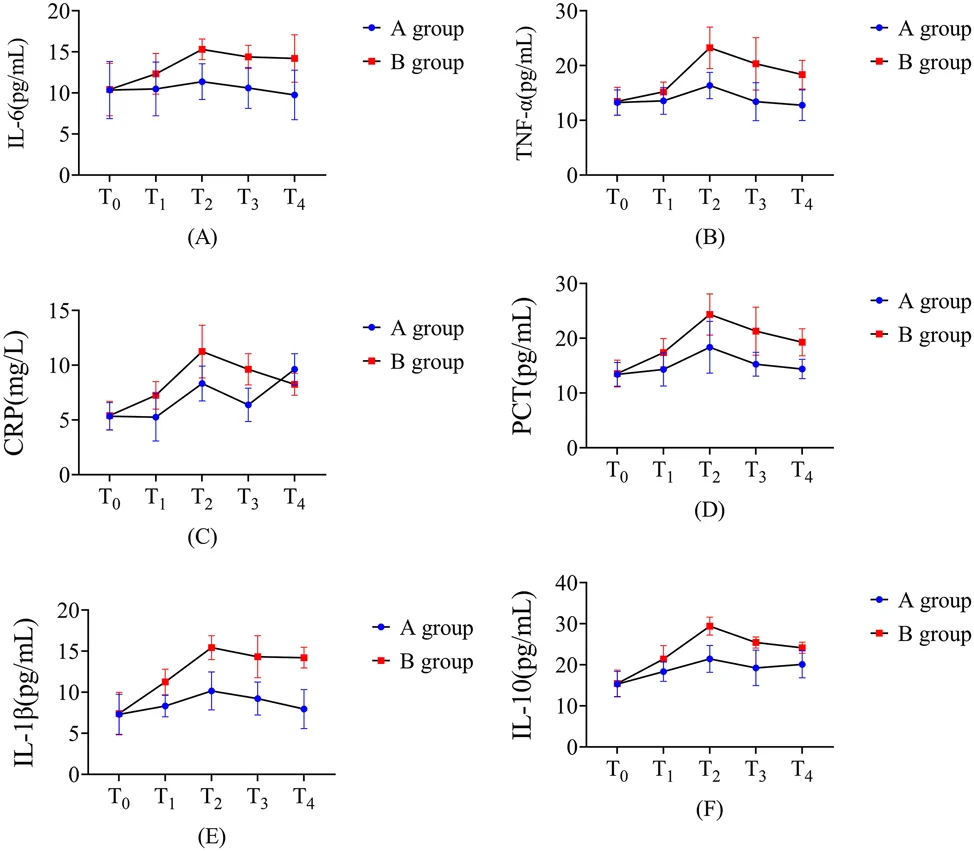
Change trend chart of immunoinflammatory reaction indicator level in the two groups. (A)–(F) respectively represent the trend of changes in IL-6, TNF-α, CRP, PCT, IL-1β, and IL-10 levels. Baseline (*T*_0_), 10 min after induction (*T*_1_), 10 min after maintenance (*T*_2_), 10 min before the end of surgery (*T*_3_), and at the end of surgery (*T*_4_).

From *T*_0_ to *T*_4_, the concentrations of IL-6, TNF-α, CRP, PCT, IL-1β, and IL-10 in Group A remained generally lower than those observed in the Group B. During this period, the fluctuations of these biomarkers in Group A were marked, indicative of significant pharmacological effects that normalized their levels to those commonly found in surgical environments. In contrast, the biomarker levels in Group B showed minimal variability, although some values did not fall within the anticipated range. Significant disparities in these inflammatory markers were evident between the two groups throughout *T*_0_ to *T*_4_, as depicted in Figure 4.

### 3.4 Comparison of sedation score across both groups

Throughout the *T*_3_ and *T*_4_ periods, Ramsay Sedation Scale (RSS) scores for Group A were lower than those recorded for the Group B (*P* < 0.01). For comprehensive data, see Table 4.

**Table 4:** Comparison of the RSS across both groups (*x̄* ± *s*, score).

Group	*n*	Conscious state		Breathing		Circulating situation	
		*T*_3_	*T*_4_	*T*_3_	*T*_4_	*T*_3_	*T*_4_
A group	50	2.09 ± 0.47	1.89 ± 0.31	1.86 ± 0.23	1.40 ± 0.19	2.14 ± 0.34	1.38 ± 0.26
B group	50	2.56 ± 0.23	2.02 ± 0.28	2.01 ± 0.33	1.76 ± 0.31	2.49 ± 0.13	1.79 ± 0.35
*t*		6.351	2.201	2.637	7.001	6.799	6.649
*P*		<0.001	0.030	0.010	<0.001	<0.001	<0.001

**Group**	***n***	**Reflection case**		**Muscle tonus**		**Pain stimulation response**	
		***T*_3_**	***T*_4_**	***T*_3_**	***T*_4_**	***T*_3_**	***T*_4_**

A group	50	2.34 ± 0.15	0.90 ± 0.23	2.34 ± 0.23	1.23 ± 0.38	1.03 ± 0.25	0.97 ± 0.16
B group	50	2.76 ± 0.12	1.25 ± 0.14	2.59 ± 0.14	1.56 ± 0.41	1.25 ± 0.10	1.10 ± 0.20
*t*		15.460	9.191	6.565	4.174	5.777	3.589
*P*		<0.001	<0.001	<0.001	<0.001	<0.001	0.001

10 min before the end of surgery (*T*_3_), and at the end of surgery (*T*_4_).

**Figure 5:**
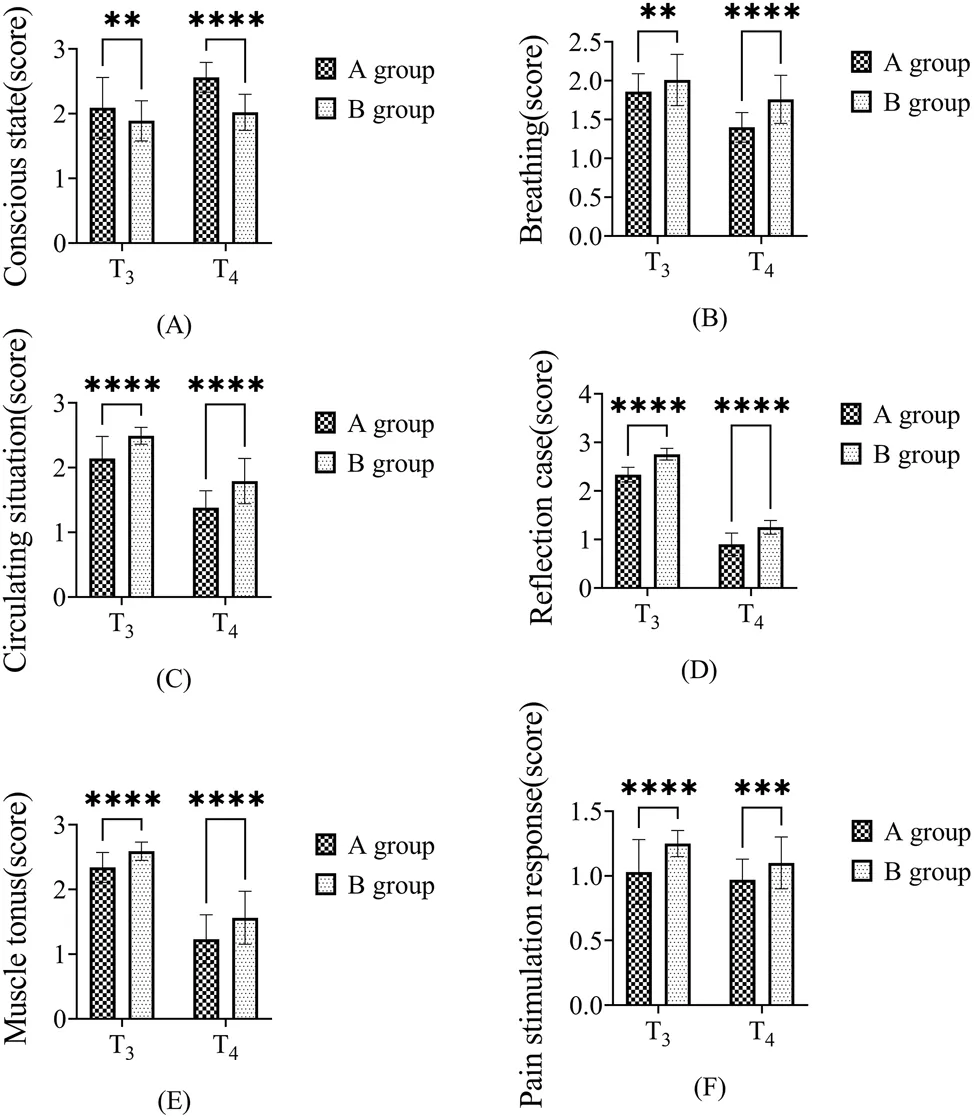
Histogram of sedation score in the two groups. (A)–(F) correspond to the comparison of consciousness, respiration, circulation, reflex, muscle tension (1–3 points), and pain stimulation reaction scores respectively; the *X* axis represents the time point, i.e. 10 min before the end of surgery (*T*_3_), and at the end of surgery (*T*_4_); the *Y* axis represents the indicator level range; the marker line represents the median range; “**” and “***” indicates 0.05 > *P* > 0.001, and “****” indicates *P* < 0.001.

At the *T*_3_ and *T*_4_ time points, RSS scores for Group A were significantly lower compared to the Group B, with statistical significance confirmed (*P* < 0.05). For further details, refer to Figure 5.

### 3.5 Comparative analysis of adverse reaction rates between the two groups

From *T*_0_ to *T*_4_, Group A displayed a reduced incidence of adverse reactions in comparison to the Group B (*P* < 0.05). Detailed information is available in Table 5.

**Table 5:** Comparison of adverse reactions rate across both groups [*n* (%)].

Group	*n*	Respiratory depression	Nausea and vomitting	Hypopiesia	Other	Total incidence
A group	50	2 (4.00)	3 (6.00)	1 (2.00)	0 (0.00)	6 (12.00)
B group	50	3 (6.00)	2 (4.00)	6 (12.00)	3 (6.00)	14 (28.00)
*t*						4.000
*P*						0.046

**Figure 6:**
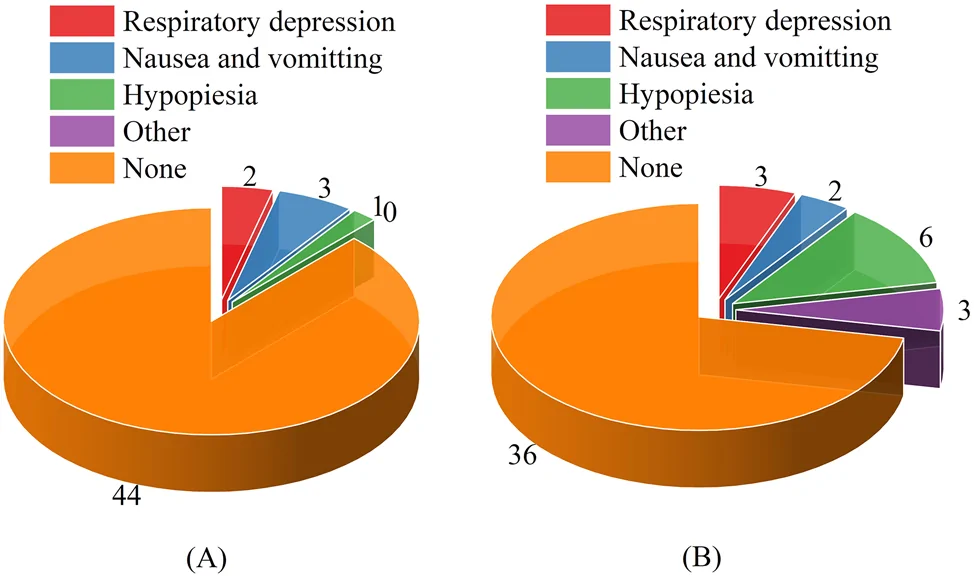
Pie distribution diagram of adverse reactions rate across both groups. (A)–(B) correspond to the proportion of adverse reaction incidence in all patients for both groups, respectively; where red represents respiratory depression, blue represents nausea and vomiting, green represents hypotension, purple represents other adverse reactions, and yellow represents no adverse reaction.

Throughout the *T*_0_ to *T*_4_ interval, Group A demonstrated a notably reduced frequency of adverse reactions when compared with Group B. Additional details can be found in Figure 6.

## 4 Discussion

With the rapid increase in the number of AF patients, RFCA is increasingly widely used as an important medical treatment for patients with this disease. More clinical researchers begin to pay more attention to the key elements to improve the efficacy of surgical treatment [27]. As a key link during surgery, excellent anesthesia effect is of great clinical significance for maintaining stable vital signs and reducing patient stress response [28], and the choice of a more appropriate anesthetic has far-reaching implications. This study primarily focused on exploring the unique effects of anesthesia administered through a combination of dexmedetomidine and remifentanil on patients with atrial fibrillation who were undergoing radiofrequency catheter ablation.

The results of this research suggest a preliminary conclusion that the combined administration of dexmedetomidine and remifentanil as an anesthetic significantly enhances hemodynamic stability and blood flow regulation in patients receiving RFCA for atrial fibrillation. In particular, dexmedetomidine interacts with α_2_-adrenergic receptors in the central nervous system, which leads to a reduction in norepinephrine release [29], consequently lowering neural excitability [30]. In this basis, it affects the peripheral vascular system and inhibits vascular smooth muscle contraction while reducing the release of angiotensin II and aldosterone [31], Bidirectional relaxation of vascular smooth muscle [32] can comprehensively improve patients’ hemodynamic indicators such as MAP, HR and blood pressure. In addition, it has been pointed out [33] that the changes in CI and CO levels are significantly associated with HR and blood pressure levels, while dexmedetomidine can improve the changes of patients’ HR and blood pressure levels, reduce myocardial oxygen consumption, improve myocardial perfusion, strengthen and protect cardiac function [34], thus improving CI and CO levels. In addition, in combination with the analgesic effect of remifentanil, it can inhibit sympathetic nerve activity [35] and comprehensively improve the hemodynamic indicator level of patients treated with RFCA.

Additionally, the combination of dexmedetomidine and remifentanil anesthesia shows potential benefits in improving the levels of immunoinflammatory response indicators in patients undergoing RFCA treatment. Dexmedetomidine, a key α_2_-adrenergic receptor agonist, efficiently suppresses the activation of inflammatory pathways including NF-κB [36], diminishes immune cell activation [37], curtails the discharge of inflammatory mediators [38], and regulates overall immune function [39]. Coupled with the analgesic effect of remifentanil, it effectively alleviates patients’ physiological pain, mitigating physiological stress [40]. Together, they synergistically lower the levels of inflammatory factors through bidirectional interventions of sedation and analgesia, exerting anti-inflammatory effects [41], thus effectively regulating the levels of immunoinflammatory response indicators in patients. Furthermore, dexmedetomidine combined with remifentanil anesthesia can improve patients’ sleep quality and emotional cognition by inhibiting neurotransmitters such as glutamate and gamma-aminobutyric acid [42]. This aids in maintaining sedation, reducing oxidative stress, inhibiting cell apoptosis, and protecting normal brain function [43], thereby enhancing clinical sedation outcomes. Furthermore, the concurrent administration of these agents lowers the required dose of remifentanil, which helps prevent respiratory depression characterized by reduced respiratory rate and tidal volume [44]. It also aids in muscle relaxation, decreases the likelihood of nausea and vomiting [45], and minimizes the risk of hypotension, maintaining more stable hemodynamic conditions in patients.

## 5 Conclusions

In individuals with atrial fibrillation undergoing radiofrequency catheter ablation, the combination of dexmedetomidine and remifentanil for monitored anesthesia notably improves hemodynamic stability, reduces inflammatory markers, enhances the quality of sedation, and diminishes the occurrence of adverse reactions when compared to the sole use of remifentanil. However, the study’s findings are potentially limited by factors such as its small sample size and a possible lack of diversity, which may hinder the broader applicability of the results. Future studies should expand the scope of participants to better assess the anesthetic effects comprehensively. Additionally, the research does not account for all possible influencing factors, including medication intake and lifestyle habits, which could distort the outcomes and diminish their reliability. Therefore, it is advisable to implement a multi-center study design with a larger sample to improve sample representativeness and enhance the reliability and generalizability of the findings. Simultaneously, incorporating more biomarkers would provide a deeper understanding of the biological impacts of anesthesia on patient physiology and offer more foundational data for clinical application, integrating pathologic and molecular level analyses.
